# Detecting and characterizing copy number variation in a large commercial U.S. Holstein cattle population

**DOI:** 10.1186/s12864-025-11536-7

**Published:** 2025-04-16

**Authors:** Giovanni C. Ladeira, Pablo J. Pinedo, José E. P. Santos, William W. Thatcher, Fernanda M. Rezende

**Affiliations:** 1 Department of Animal Sciences, University of Florida, 2250 Shealy Drive, PO Box 110910, Gainesville, FL 32611 USA; 2 Department of Animal Sciences, Colorado State University, Fort Collins, CO USA

**Keywords:** Dairy cattle, Enrichment analysis, Genome-wide mapping, Structural variation

## Abstract

**Background:**

Copy number variations (CNVs) are an important source of genomic variation that play an active role in modulating biological processes by altering gene expression and dosage. These structural variants involve duplications and deletions of segments usually exceeding 1 kilobase in size, dispersed throughout the genome of humans and livestock individuals. We mapped CNVs from high-density single-nucleotide polymorphism (SNP) genotyping array data on 3,601 Holsteins. Following, we explored their association with reported quantitative trait loci (QTLs), genes, and biological pathways, unveiling the potential biological contributions of CNVs to economically important traits in the dairy industry and breeding programs.

**Results:**

We identified 4,113 non-redundant high-confidence CNVs, of which 78% were deletions and 22% duplications, distributed across all bovine autosomal chromosomes (BTA). Out of the 1,184 compiled CNV regions (CNVRs) covering 3.02% of the autosomal genome, 199 novel CNVRs were mapped. QTLs overlapping with CNVRs detected in this study were enriched for 140 economically important traits, such as milk yield, conception and pregnancy rates, susceptibility to diseases and length of productive life, indicating that CNVs likely underlie productive, reproductive and health performance in Holstein dairy cattle. Moreover, detected CNVRs overlapped with 2,788 annotated genes, including those linked to milk production, fertility, and immune response in cattle, such as *DGAT1*, *AFF1*, and *ADAMTS13* genes. Furthermore, the gene set analysis revealed GO terms related to metabolic processes, immune system, response to stimulus, and cellular binding activities. Notably, enriched GO terms highlighted relevant genes to cattle health and reproduction overlapping CNVRs, such as *DEFB4A*, *GATA3*, *GNB1*,* and PPP1R1B*.

**Conclusions:**

We mapped and demonstrated the characteristics of genome-wide distributed CNVs in a large commercial Holstein population genotyped with a high-density SNP array. Collectively, the results emphasize the role of CNVs as a valuable resource of genetic variation and contribute to better understand the genetic architecture of economic complex traits in dairy cattle. Furthermore, these findings may provide opportunities for the development of novel and enhanced genomic selection strategies in Holstein cattle.

**Supplementary Information:**

The online version contains supplementary material available at 10.1186/s12864-025-11536-7.

## Background

Over the last decade, the U.S. dairy industry has experienced a robust 8.4% growth in mean individual cow milk yield [1], which has more than offset the relatively small 1.3% rise in cow numbers [2]. The productivity boost in dairy operations resulted from a multifaceted approach, including advances in nutrition, reproduction, management, and genetics, with the latter contributing to more than 60% of the gain over the past 47 years [3]. The national genetic evaluation records highlight the steady progress in genetic merit for several production traits such as yields of milk, protein, and fat, productive life, and livability [4]. The advent of genomic technology has transformed dairy breeding programs and significantly impacted industry profits globally, mostly by reducing selection costs and enabling the selection for new traits. Since the introduction of genomic evaluations in 2009 for Holsteins and Jerseys in the U.S., the rate of genetic gain has accelerated remarkably, from $40 per year (2005–2009) to $85 per year (2010–2021) for lifetime net merit [4], mainly attributed to a substantial reduction in the generation interval, effectively doubling the rate of genetic progress. Although single nucleotide polymorphism (SNP) variations have proven valuable for genomic evaluation and understanding the genetic basis of complex traits, additional genomic variations, such as copy number variation (CNV), offer complementary insights into the genetic mechanisms influencing complex traits. By incorporating these alternative variants, we can gain a more comprehensive understanding of the genetic factors shaping economically important traits in dairy cattle.

Copy number variations are large genomic structural variants with at least 1 kilobase (kb) in size, which can be inherited or arise *de novo* and are present in a variable number of copies compared to the reference genome [5]. Copy number variation modulates both gene expression and general transcriptome patterns mainly by changing gene dosage, deleting or duplicating regulatory elements of the gene, and leading to gene interruption or fusion at the breakpoint junctions [5–7]. Thus, CNVs are recognized as an important source of genetic diversity in humans and livestock populations. Indeed, genome-wide association analyses have identified CNVs linked to key traits in dairy cattle, such as feed efficiency, milk yield and composition, and cow, daughter and heifer fertility, as well as health indicators like somatic cell score and clinical mastitis [8–12]. While these findings underscore the importance of CNVs in economically relevant traits, most association studies focused on a limited set of traits, potentially overlooking the broader contribution of detected CNVs. Therefore, conducting genome-wide mapping of CNVs and characterizing their features across multiple genomic databases can lead to a better understanding of genomic diversity and the biological processes underlying various critical traits in dairy cattle, ultimately advancing our knowledge of the functional role and importance of these variations.

Quantitative PCR (qPCR) has been employed to determine CNVs by simultaneously amplifying target regions with unknown copy numbers and reference regions with known copy numbers, allowing for relative quantification of the target loci [13]. Recently, whole genome sequencing (WGS) has become a more frequent approach for CNV detection, bringing extremely high-quality definitions of CNV boundary in livestock species [14–17]. Despite their advantages, qPCR and WGS imply greater costs compared to in silico procedures, which often restrict the animal cohorts for genome-wide variant detection studies, reducing the representativeness of population genetic diversity. In fact, the number of animals included in the WGS studies frequently ranges from tens to few hundreds in dairy cattle [14–17]. In contrast, the quality of in silico CNV calling and mapping of CNVRs depends on several factors, including genome coverage density, type of genomic information (e.g., CGH arrays, SNP arrays, low-pass or whole genome sequencing), choice of algorithm (e.g., PennCNV, QuantiSNP, and cnvPartition), and studied population size [18–22]. Altogether, finding balance between mapping precision, population representativeness, and cost feasibility has become a major challenge for CNV detection, characterization and integration into livestock selection programs.

Despite the growing number of studies on CNVs in livestock, there is still a notable gap in research that utilizes high-density genome coverage to identify and characterize CNVs in large, representative cattle populations. Frequently, studies mapping and describing CNVs from high-density SNP genotyping include a limited number of animals, usually ranging from a few dozen to fewer than a thousand [17, 23–25]. A notable exception is a study in beef cattle [26] that mapped and characterized CNVs from 3,794 Nellore individuals. This underscores the need for CNV detection in large, representative dairy cattle populations [27]. Calling CNVs from high-density SNP genotyping data in a sizable Holstein population may lead to groundbreaking genetic insights into dairy industry traits, paving the way for novel research and applications. Therefore, the main purpose of this study was to map copy number variations in a large Holstein population genotyped with high-density SNP array and subsequently characterize the detected variants based on their association with known QTLs, genes, and biological pathways.

## Materials and methods

### Sampling and genotype data

The study population comprised 3,601 Holstein individuals, including 3,387 cows from 16 herds across 7 states in the United States (California, Florida, Minnesota, New York, Ohio, Texas, and Wisconsin) plus 214 selected artificial insemination (A.I.) bulls. The cow cohort was drawn from a larger group of 11,733 females that calved between 2012 and 2014 and were enrolled in a fertility study [28]. Firstly, a subset of 2,501 cows with extreme reproductive index values within farm and calving season were genotyped [28–30]. To expand the study population, 886 randomly selected cows and 85 high-daughter pregnancy rate (DPR) and 129 low-DPR proven A.I. bulls (> 10 daughters) were genotyped. All animals were genotyped on the Illumina BovineHD BeadChip array (*n* = 777,962 SNPs; Illumina, San Diego, CA).

We updated the coordinates of SNPs from the bovine reference genome assembly UMD3.1 [31] to ARS-UCD1.2 [32] employing the information available on the National Animal Genome Research Program (NAGRP) data repository (https://www.animalgenome.org/repository/cattle/UMC_bovine_coordinates/) in an in-house pipeline. Subsequently, two subsets of genotypic data were created for CNV mapping. The first set included all 3,601 individuals and their genotypes for 720,731 autosomal SNPs with known coordinates. The second genotypic set consisted of 3,546 individuals and 705,438 autosomal SNPs with known coordinates, which passed the sample and genotype quality control (call rate ≥ 90%) performed with the QCF90 software from the BLUPF90 family of programs [33].

### Copy number variations identification and CNVR construction

We utilized both forementioned subsets of genotypic data to independently call CNVs across the Holstein genome using the PennCNV software v. 1.0.5 [34]. This software integrates several genome-wide SNP genotyping measures into a hidden Markov model, including log R ratio (LRR), B allele frequency (BAF), population frequency of B allele (PFB), and distance between two adjacent SNPs for high-resolution CNV detection. The LRR and BAF measures were inferred using the Illumina Genome Studio software package (Illumina, San Diego, CA, USA), while we estimated the PFB from BAF using the ‘compile_pfb.pl’ function. Copy number variations calling was performed with the ‘detect_cnv.pl’ function, applying the ‘-gcmodel’ option to correct LRR for genomic waves caused by guanine-cytosine (GC) content around each SNP (1 Mb window, 500 kb up and downstream) [35]. PennCNV stands out as the most reliable software for detecting CNVs from SNP data, outperforming other tools in terms of sensitivity, bias, and success rate [18]. Notably, the combination PennCNV software and BovineHD Genotyping BeadChip demonstrated high validation rates, with 91.7% for CNVs found in multiple animals and 40% for singleton CNVs, as confirmed by qPCR [24].

A three-step quality control was independently applied to both subsets of genotypic data. First, visual inspection of CNV counts revealed that animals carrying more than 1,000 CNVs strongly deviated from the population distribution (Figure S1 of Supplementary Materials 1). Second, animals were retained for further analyses if they met the following criteria: LRR standard deviation ≤ 0.30 (‘-qclrrsd 0.3’), BAF drift ≤ 0.01 (‘-qcbafdrift 0.01’), waviness factor ≤ 0.05 (‘-qcwf 0.05’) and number of CNV ≤ 1,000 (‘-qcnumcnv 1000’). Third, at the genomic structural level, CNVs were required to meet the following conditions to be included in further analyses: number of SNPs ≥ 10 (‘- numsnp 10’), length ≥ 1 kb (‘- length 1k’), and presence in at least 5 animals. A total of 3,456 animals in each subset of genotypic data passed the three-step quality control (Figure S2 of Supplementary Materials 1).

Next, we constructed high-confidence CNV regions by compiling CNVs identified in both genotypic datasets that shared the exact same start and end positions. This approach was based on the rationale that identical CNVs identified in both subsets would have higher-quality boundary definitions than those mapped in only one subset. For that, we used the ‘populationRanges’ (grl, density = 0.1) function from the CNVRanger R/Bioconductor package [35] to merge overlapping CNVs, defined as those sharing at least 1 base pair, into unified CNVR. To minimize false positive regions potentially introduced by extremely long CNVs, we trimmed segments covered by less than 10% of the contributing CNVs within a CNVR. CNVRs were classified into deletion, duplication, and complex regions if all CNVs within the CNVR were classified as deletion, duplication, or deletion and duplication, respectively. The ggplot2 package [36] was employed to generate a visual representation of the high-confidence CNVRs on a chromosome map. Lastly, we cross-referenced our compiled CNVRs with the Ensembl structure variation database (Cow release 109) [37] and classified as novel CNVRs those that lacked overlap with annotated structural variations.

### Functional impact of CNVRs

Quantitative trait locus (QTL) and gene annotation were performed using the R software. This involved overlapping high-confidence CNVR coordinates (chromosome, start, and end positions) with QTLs coordinates from the Animal QTL database (release 50) [38] and gene coordinates from the Ensembl database [37], both mapped to the ARS-UCD1.2 bovine genome assembly.

Quantitative trait locus enrichment analyses were performed to test the genome-wide representativeness of trait-specific QTLs overlapping CNVRs. The number of CNVR-overlapping QTLs associated with a specific trait was compared to the total number of QTLs associated with that specific trait in cattle. Subsequently, this information was integrated into Fisher’s exact test [39] to estimate whether the QTLs associated with each trait overlapped with CNVRs at a frequency greater than expected by chance [40]. The *P*-value of observing *k* QTLs associated with a specific trait overlapping with CNVRs was calculated by

$$\:P-\text{v}\text{a}\text{l}\text{u}\text{e}=1-\sum\:_{i=0}^{k-1}\frac{\left(\genfrac{}{}{0pt}{}{S}{i}\right)\:\left(\genfrac{}{}{0pt}{}{N-S}{m-i}\right)}{\left(\genfrac{}{}{0pt}{}{N}{m}\right)}$$

where $$S$$$$\:N$$ is the total number of QTLs analyzed in the study, and $$\:m$$ is the number of QTLs overlapping CNVRs. Then, the False Discovery Rate (FDR), as calculated by the Benjamini-Hochberg procedure [41], was applied to correct the *P*-value (*P*_FDR_). The *P*-value corrected for false discovery was used to determine, based on an alpha level of 5%, if the number of QTLs associated with each trait overlapped with CNVRs was larger than the number of QTLs expected, by chance, to overlap with CNVRs. Therefore, the QTLs overlapped with CNVRs were considered enriched for traits when *P*_FDR_ < 0.05.

The Ensembl Variant Effect Predictor (VEP) [42] (Cow release 109) was used to predict the molecular consequences that each CNVR may have on each transcript and explore their functional impact. In addition, the gene set enrichment analysis was implemented using the overrepresentation test of the PANTHER software [43, 44]. For that, the number of genes overlapped with CNVRs and underlying each Biological Process, Cellular Component, or Molecular Function was compared with the total number of genes underlying each Biological Process, Cellular Component, or Molecular Function available in the Ensembl database following the same statistical approach used for the QTL enrichment analysis.

## Results

### CNV calls in U.S. Holsteins

Two genotypic data subsets, differing in sample size and number of SNP markers due to quality control, were used for CNV calling. The first dataset, without quality control, consisted of 3,601 animals with 720,731 SNP genotypes. This yielded 73,422 CNVs mapped, representing 4,631 non-redundant CNVs in 3,465 animals. In contrast, the dataset with quality control comprised 3,546 animals genotyped for 705,438 SNPs, resulting in 71,993 CNVs mapped, denoting 4,518 non-redundant CNVs in 3,465 animals. Notably, 68,982 total CNVs, conveying 4,113 non-redundant CNVs (Table S1 of the Supplementary Materials 2), were identified in animals present in both genotypic datasets with identical start and end chromosomal positions, hereby termed high-confidence CNVs. For clarity, “total CNVs” refers to the cumulative count of all mapped high-confidence CNVs in the studied population, whereas “non-redundant CNVs” indicates the number of high-confidence CNVs with distinct start and/or end chromosomal coordinates mapped in this Holstein population.

**Fig. 1:**
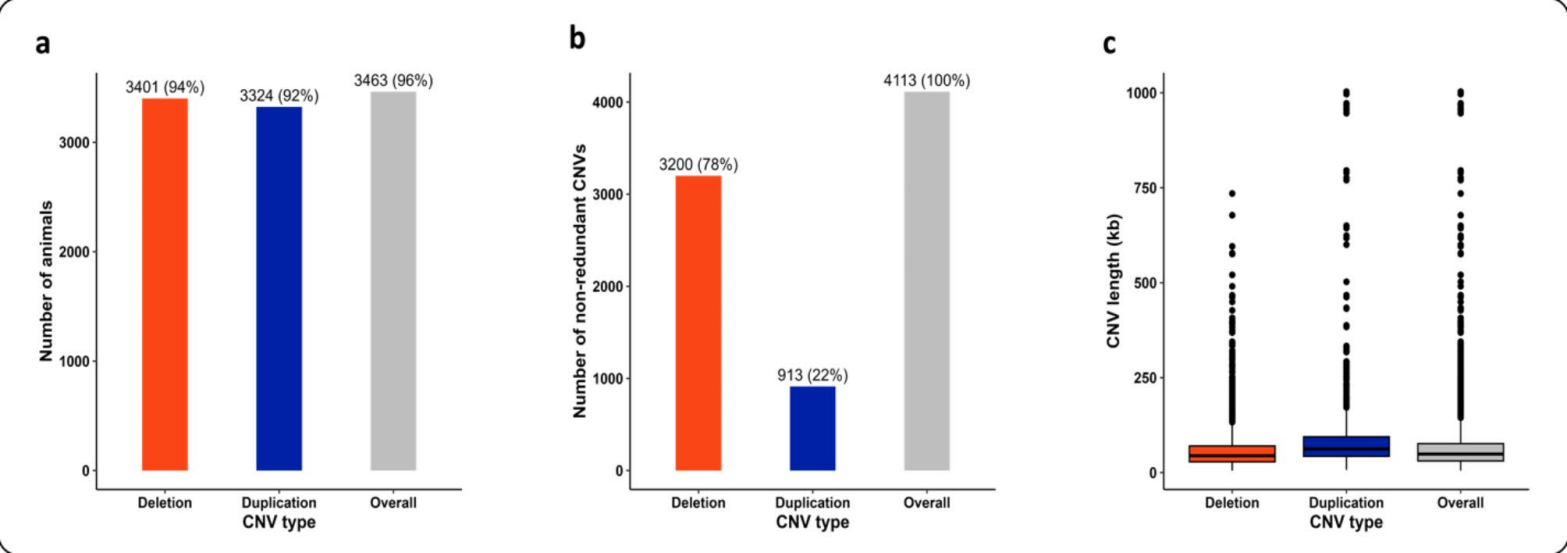
Distributions of non-redundant high-confidence CNVs by type. **(a)** Number of animals carrying at least one deletion (red bar), at least one duplication (blue bar) and at least one deletion or duplication (gray bar), followed by the percentage out of all genotyped animals in parentheses. **(b)** Absolute frequency of non-redundant high-confidence CNVs by type, followed by the percentage out of non-redundant high-confidence CNVs in parentheses. **(c)** Boxplot distribution of non-redundant high-confidence CNV lengths by type

The total high-confidence CNVs comprised 50,061 deletions (72.57%) and 18,921 duplications (27.43%) identified in 3,463 individuals, which represent 96% of all genotyped animals (Fig. 1a). It is noteworthy that two animals from each genotypic subset were excluded from the high-confidence subset due to carrying CNVs that were not reciprocally mapped in the other subset. The number of CNVs per animal ranged from 1 to 222 (Figure S3 of Supplementary Materials 1), with a mean of 19.92. The absolute frequency of each CNV in the considered Holstein population ranged from 5 to 1,005 animals carrying the respective variant, averaging 16.77 animals carrying a specific CNV. The minimum, maximum, and mean lengths of total high-confidence CNVs were 5.44 kb, 1,002.92 kb, and 81.50 kb, respectively. Deletions had a mean length of 65.05 kb, whereas duplications averaged 125.03 kb. Notably, deletions were 47.97% smaller and 2.64 times more frequent than duplications.

Figure 1 displays the number of animals carrying CNVs, the frequency and length distribution of non-redundant high-confidence CNVs by type. The non-redundant high-confidence CNVs consisted of 3,200 deletions (77.80%) and 913 duplications (22.20%), dispersed across the 29 bovine autosomal chromosomes (BTAs). Figure S4 of the Supplementary Materials 1 illustrates the absolute frequency of CNV by chromosome. On average 141.82 non-redundant CNVs were mapped per chromosome, ranging from 43 CNVs on BTA28 to 265 CNVs on BTA19. Table S2 of the Supplementary Materials 2 provides additional descriptive statistics for non-redundant high-confidence CNVs. The mean length of non-redundant high-confidence CNVs was 67.21 kb. Markedly, deletions (average length: 59.94 kb) were 35.38% smaller and 3.50 times more frequent than duplications (average length: 92.70 kb). The average probe density, defined as the number of supporting SNPs per Mb of CNV, was 299.55 SNPs/Mb.

### Compiled CNVRs

**Fig. 2:**
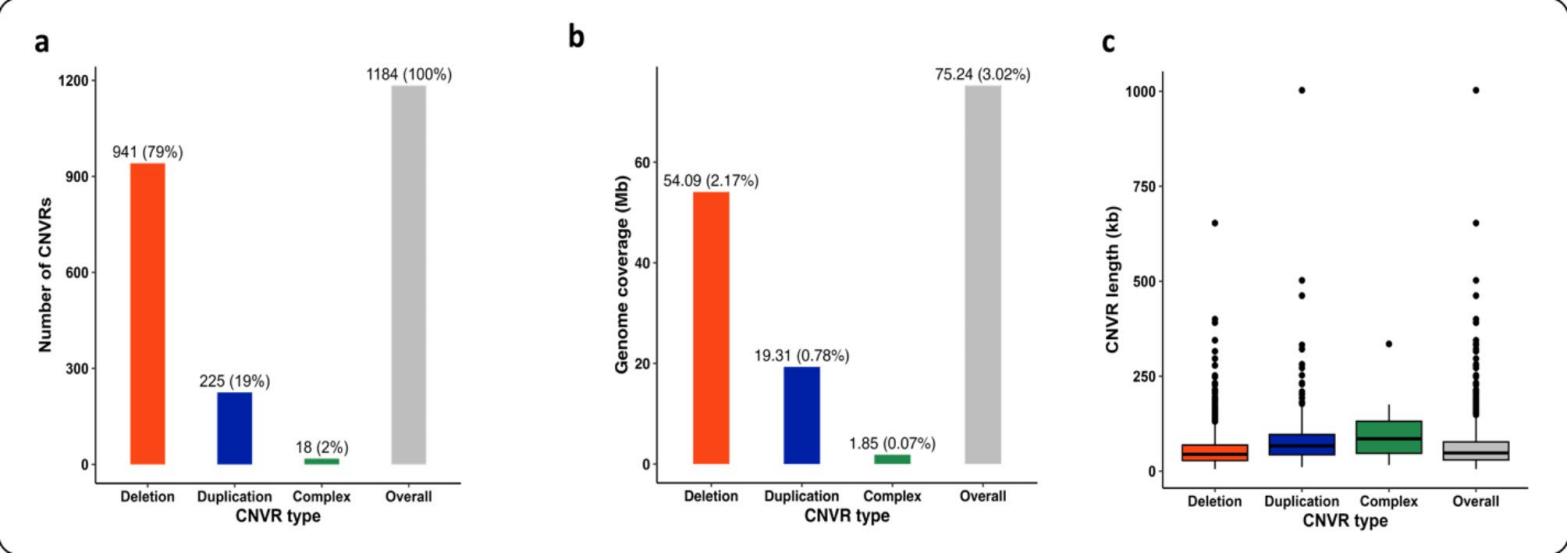
Distributions of high-confidence CNVRs by type. **(a)** Absolute frequency of CNVRs by type, followed by the percentage out of the total number of CNVRs in parentheses. **(b)** Autosome genome covered length by CNVR type, followed by the percentage of bovine autosomal chromosome covered (ARS-UCD1.2) in parentheses. **(c)** Boxplot distribution of CNVR lengths by type

High-confidence copy number variation regions (CNVRs) were constructed from high-confidence CNVs, which were identified in animals present in both genotypic datasets and shared identical coordinates. We compiled 1,184 high-confidence CNVRs, covering 3.02% of the bovine autosomal genome (i.e., 75.24/2489.39 Mb). Figure 2 illustrates the frequency, autosome genome coverage, and length distribution (boxplot) of high-confidence CNVRs by type. As anticipated, deletion regions outnumbered duplication regions by approximately fourfold, reflecting the greater frequency of CNV deletions compared to duplications. Moreover, deletion regions spanned more than twice the genomic length of duplication regions. Copy number variation regions of complex type represent genomic regions where some animals exhibit deletions, whereas others exhibit duplications. Although these complex CNVRs occur less frequently and cover a smaller genomic portion than deletions and duplications, they highlight regions exhibiting pronounced copy number variability within the population. Supplementary Materials 2 provides detailed information, including mapped high-confidence CNVRs, CNVRs distribution by length, and descriptive statistics for CNVRs, presented in Tables S3, S4, and S5, respectively. The CNVR length distribution revealed that 97.47% were 200 kb or shorter, 2.11% were larger than 200 kb but 400 kb or shorter, 0.25% were larger than 400 kb but 600 kb or shorter, and 0.17% were larger than 600 kb.

**Fig. 3:**
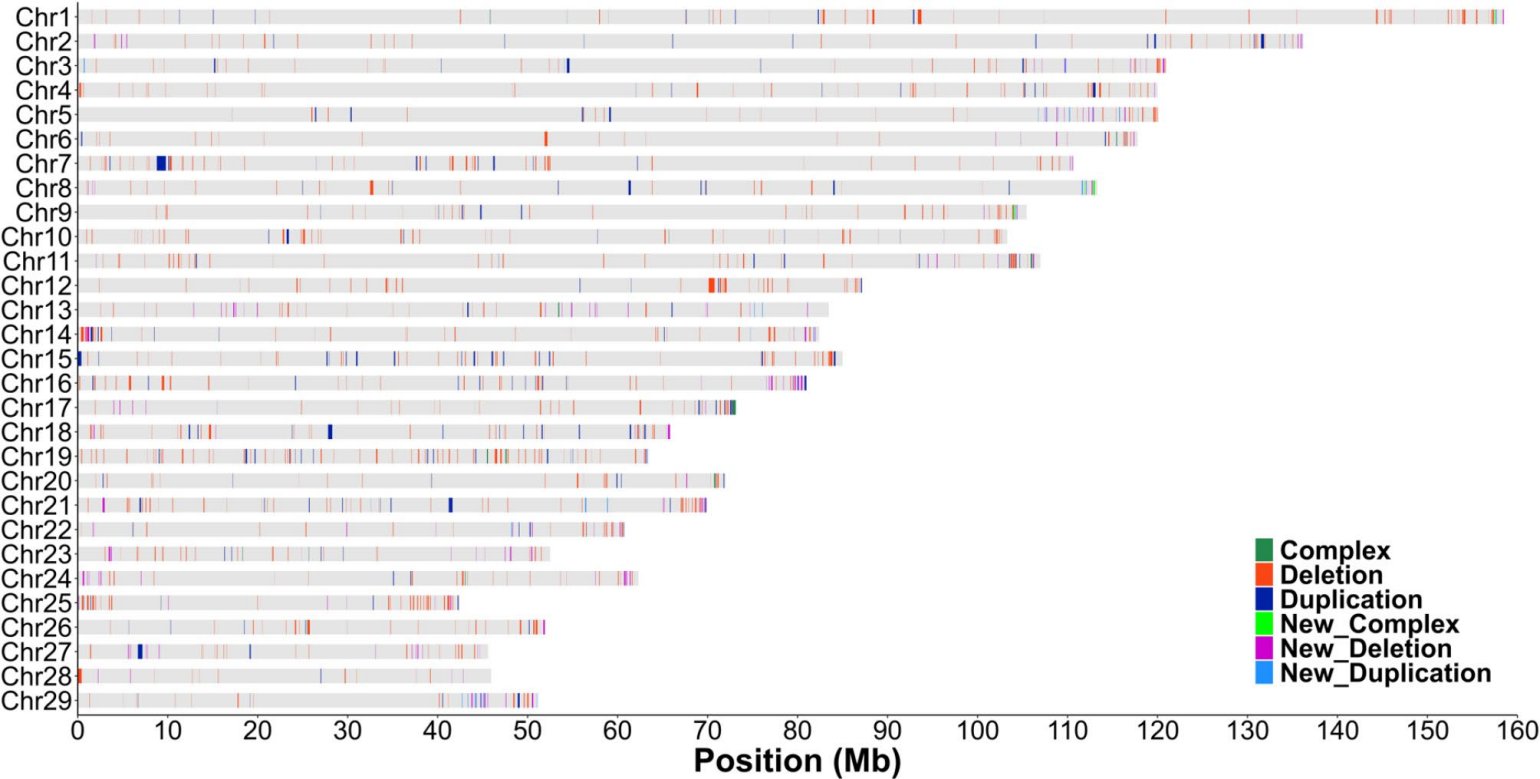
CNVR map of a representative Holstein commercial population. The horizontal gray bars represent the 29 bovine autosomal chromosomes, with chromosome coordinates indicated on the x-axis. The legend illustrates the CNVR types: complex (dark green), deletion (orange), duplication (dark blue), new complex (light green), new deletion (pink), and new duplication (light blue)

Figure 3 displays the CNVR map, illustrating the genomic distribution of CNVs in this Holstein population. Despite their widespread presence across the autosomal genome, CNVRs showed a non-uniform chromosome-wide distribution, with a tendency to occur at chromosomal extremes. Supplementary Materials 2 Table S6 provides detailed information on high-confidence CNVRs per chromosome. The number of CNVRs per chromosome varied from 14 to 67, with coverage ranging from 1.42% on BTA6 to 7.08% on BTA19.

Remarkably, 881 compiled CNVRs (74.41%) had at least 50% of their length overlapping with previously annotated structural variations in the bovine genome. In contrast, 104 CNVRs (8.78%) had less than 50% overlap with previously reported structural variations. Furthermore, we discovered 199 new CNVRs, accounting for 16.81% of the total identified CNVRs, which did not overlap any base pair with existing structural variations annotated in the Ensembl database. Tables S7 and S8 of Supplementary Materials 2 show the percentage length of each CNVR overlapping with annotated structural variations and the newly reported CNVRs, respectively.

### Functional impact of CNVRs

**Fig. 4:**
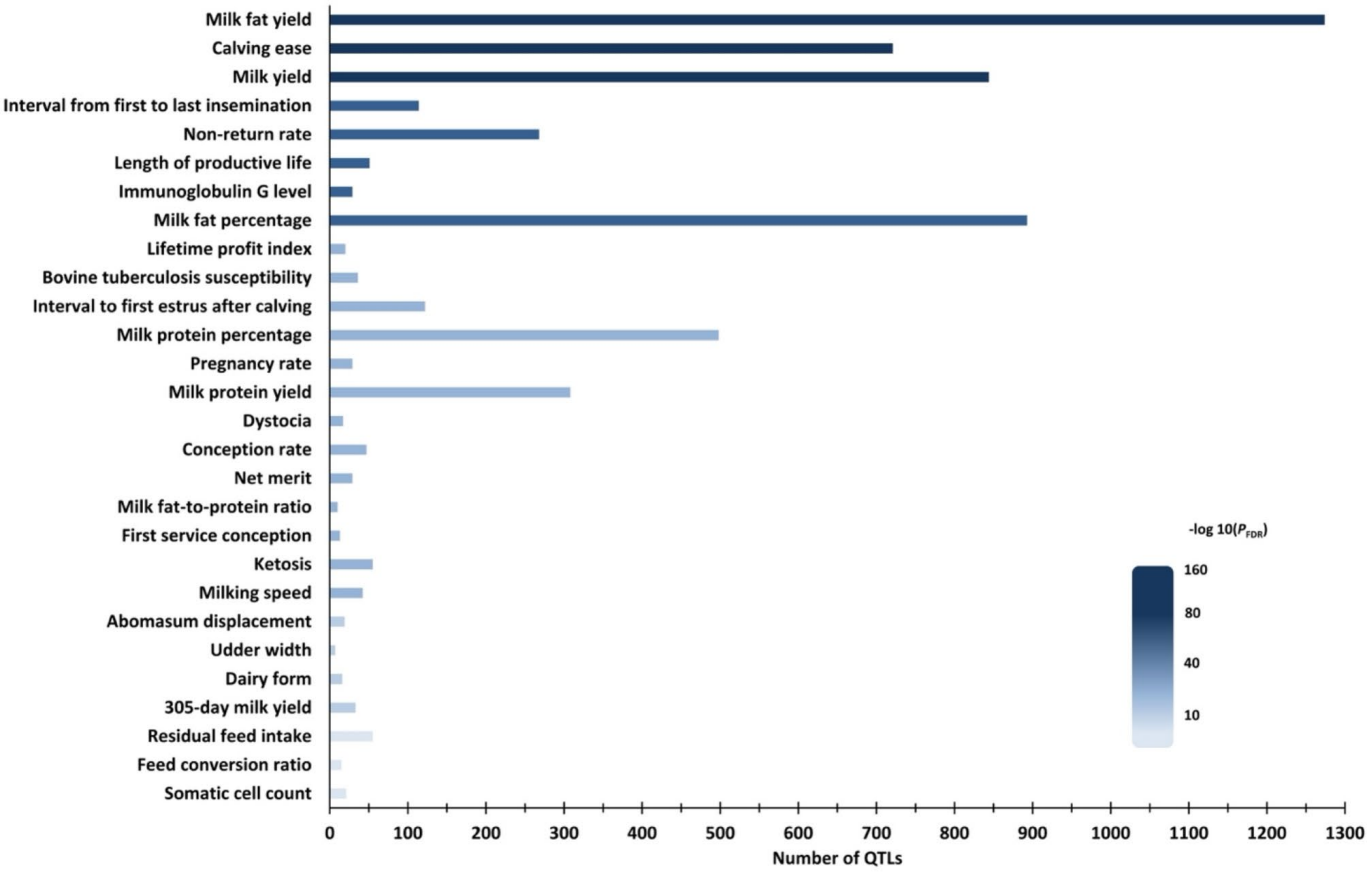
Selected enriched traits previously associated with QTLs overlapped with CNVRs. The x-axis shows the number of QTLs overlapping CNVRs that are associated with traits indicated on the y-axis. The shades of blue indicate the adjusted *P*-value (*P*_FDR_), where the darker the blue, the smaller the *P*_FDR_

Tables S9 and S10 of Supplementary Materials 2 list all 135,203 QTLs considered for annotation and the 20,139 annotated QTLs overlapping CNVRs, respectively. Among 1,184 mapped high-confidence CNVRs, 1,167 CNVRs overlapped 10,337 distinct QTLs reported in cattle, with some QTLs covered by more than one CNVR, representing 365 QTL traits. Notably, 54.93% of these QTLs were classified under QTL type milk (5,678 QTLs), 15.77% as reproduction (1,630 QTLs), 11.70% as meat and carcass (1,209 QTLs), 7.93% as production indices (820 QTLs), 5.24% as exterior (542 QTLs), and 4.43% as health (458 QTLs). Figure 4 illustrates selected enriched traits identified in the QTL enrichment analysis. This analysis revealed that QTLs overlapped with CNVRs were enriched for 140 distinct traits. Milk traits comprised 30.71% of the enriched traits (43 traits), including milk yield, milk solids yield, milking speed and several milk components content. Meat and carcass traits accounted for 23.57% and consisted of 33 traits, with many related to fat deposition and composition. The health group represented 15.71% with 22 enriched traits, such as immune globulin G level, ketosis, abomasum displacement, and somatic cell count. Comprising 11.43% with 16 traits, reproductive traits included calving ease, non-return rate, and conception and pregnancy rates. Production indices made up 10% with 14 traits, encompassing the Canadian lifetime profit index, lifetime net merit in the U.S., and feed efficiency measures. Finally, exterior constituted 8.57% with 12 traits, such as udder width, dairy form and feet and leg conformation. A comprehensive list of traits included in QTL enrichment analysis is provided in Table S11 of Supplementary Materials 2. Notably, QTLs associated with economically pivotal dairy traits overlapped with CNVRs in a coordinated manner, beyond just randomness.

The gene annotation revealed that 1,043 CNVRs overlapped with 2,788 genes from the Ensembl database, with some genes overlapped by more than one CNVR. Of these, 61.22% (1,707 genes) were completely overlapped with CNVRs, while 38.78% (1,081 genes) were partially overlapped. Table S12 of Supplementary Materials 2 presents the comprehensive list of genes overlapped with CNVRs. Protein-coding genes comprise 87.34% (2,435) of the total genes spanned by CNVRs. Complementarily, analysis of molecular consequences of CNVRs revealed that 19% of CNVRs are intron variants, whereas 18% are transcription ablation, affecting transcript features. Additionally, 17% affect coding sequences by changing their composition, and 15% result in feature truncation, reducing genomic features in relation to a reference sequence. Furthermore, 9% of CNVRs are located at 3′-untranslated regions, and 8% are stop loss, altering terminator codons. Moreover, 7% are located at 5′-untranslated regions, and 3% are transcription amplification, leading to the amplification of a region containing a transcript. The remaining 4% have other molecular functions. A detailed explanation of the molecular consequence terms (Sequence Ontology terms) can be found on the Ensembl web page (https://useast.ensembl.org/info/genome/variation/prediction/predicted_data.html#consequences). Hence, CNVs are distributed across functional genomic segments, highlighting their significance in genomic variation.

Gene set analysis exposed 31 overrepresented GO terms for Biological Processes, 18 for Cellular Component, and 7 for Molecular Function, as presented in Table 1. The Biological Process terms describe large processes accomplished by multiple molecular activities, whereas the Cellular Component terms describe a location relative to cellular compartments and structures occupied by a macromolecular machine, and the Molecular Function terms describe activities that occur at the molecular level. Markedly, GO terms for Biological Process describing metabolic processes (GO:0071704, GO:0008152, GO:0044238, GO:0006807, GO:0044237, GO:0043170, GO:1901564, GO:0006725, GO:0046483, and GO:0006139), response to stimulus (GO:0050896, GO:0051716, GO:0007165, GO:0009607, GO:0023052, GO:0043207, GO:0051707, GO:0007154, and GO:0080134), and immune response (GO:0006952, GO:0098542, GO:0098542, and GO:0002376) were frequently associated with genes overlapped with CNVRs. GO terms for Cellular Component indicated high presence of macromolecular machines associated with genes potentially affected by CNVRs located at cell structure (GO:0110165, GO:0043227, GO:0005622, GO:0043231, GO:0016020, GO:0012505, and GO:0031974) and organelles (GO:0043226, GO:0043229, GO:0070013, and GO:0043233). GO terms for Molecular Function indicated that genes overlapped with CNVRs considered in the gene set analysis play a role in cellular binding activities (GO:0005488, GO:0097159, GO:0005515, and GO:1901363). All these GO terms expose the potential impacts of CNVRs in several molecular activities.

**Table 1:** Gene ontology (GO) enriched categories for biological process, cellular component, and molecular function based on genes overlapped with all compiled CNVRs

GO ID	Description	*P*_(FDR)_
**Biological process**		
GO:0008150	biological process	4.91E-18
GO:0009987	cellular process	1.62E-14
GO:0050896	response to stimulus	9.63E-07
GO:0071704	organic substance metabolic process	2.16E-06
GO:0008152	metabolic process	6.58E-06
GO:0051716	cellular response to stimulus	9.07E-06
GO:0044238	primary metabolic process	1.33E-05
GO:0065007	biological regulation	3.10E-05
GO:0006807	nitrogen compound metabolic process	4.85E-05
GO:0050789	regulation of biological process	5.67E-05
GO:0044237	cellular metabolic process	7.25E-05
GO:0006950	response to stress	7.69E-05
GO:0050794	regulation of cellular process	0.0004
GO:0043170	macromolecule metabolic process	0.0012
GO:1901564	organonitrogen compound metabolic process	0.0022
GO:0034641	cellular nitrogen compound metabolic process	0.0049
GO:1901360	organic cyclic compound metabolic process	0.0119
GO:0006952	defense response	0.0120
GO:0006725	cellular aromatic compound metabolic process	0.0141
GO:0007165	signal transduction	0.0145
GO:0009607	response to biotic stimulus	0.0188
GO:0023052	signaling	0.0217
GO:0032501	multicellular organismal process	0.0278
GO:0043207	response to external biotic stimulus	0.0280
GO:0051707	response to other organism	0.0287
GO:0007154	cell communication	0.0305
GO:0080134	regulation of response to stress	0.0353
GO:0046483	heterocycle metabolic process	0.0374
GO:0098542	defense response to other organism	0.0425
GO:0006139	nucleobase-containing compound metabolic process	0.0440
GO:0002376	immune system process	0.0448
***Cellular component***		
GO:0110165	cellular anatomical entity	2.45E-20
GO:0005575	cellular component	5.30E-20
GO:0043227	membrane-bounded organelle	3.57E-12
GO:0005622	intracellular anatomical structure	3.69E-12
GO:0043226	organelle	1.34E-11
GO:0043229	intracellular organelle	6.78E-11
GO:0043231	intracellular membrane-bounded organelle	9.60E-11
GO:0005737	cytoplasm	2.14E-09
GO:0005576	extracellular region	1.64E-07
GO:0016020	membrane	2.44E-06
GO:0005615	extracellular space	3.37E-05
GO:0032991	protein-containing complex	0.0003
GO:0005634	nucleus	0.0011
GO:0071944	cell periphery	0.0143
GO:0012505	endomembrane system	0.0197
GO:0031974	membrane-enclosed lumen	0.0204
GO:0070013	intracellular organelle lumen	0.0216
GO:0043233	organelle lumen	0.0230
***Molecular function***		
GO:0003674	molecular function	2.42E-14
GO:0005488	binding	5.35E-10
GO:0003824	catalytic activity	0.0009
GO:0097159	organic cyclic compound binding	0.0125
GO:0004888	transmembrane signaling receptor activity	0.0139
GO:0005515	protein binding	0.0153
GO:1901363	heterocyclic compound binding	0.0255

## Discussion

We reported CNVs calling from high-density SNP arrays (777k) in a large, representative Holstein population (*n* = 3,601), characterizing the features of mapped CNVs and revealing their links with QTLs, genes, and biological mechanisms. Notably, we identified CNV regions, including 199 novel CNVRs, spanning the entire bovine autosome genome. These CNVRs overlapped QTLs associated with milk yield, milk components, reproductive and health traits in cattle, occurring at frequencies greater than expected by chance. Moreover, mapped CNVRs overlapped 2,788 genes and several functional non-genic regions, potentially modulating gene expression through gene dosage changes and/or alterations in DNA fragments influencing transcription. The enrichment analysis revealed these genes contribute to biological functions underlying metabolic processes, response to stimulus, and immune response. These findings demonstrate that CNVs span functional genomic regions, underpinning economically important polygenic traits in dairy cattle, including fat yield, calving ease, and milk yield. Consequently, this study enhances our understanding of CNV roles in the genetic variation of complex traits.

Deletions were more frequent and smaller than duplications among 4,113 non-redundant high-confidence CNVs mapped. These CNVs were distributed across all autosome chromosomes, supporting their role in polygenic traits. Copy number variations arise from three major mechanisms: nonallelic homologous recombination (NAHR), nonhomologous end joining (NHEJ), and fork stalling and template switching (FoSTeS) [45–48]. These mechanisms occur in different frequencies throughout the genome, reflecting the uneven genome-wide and chromosomal-wide distribution of CNVs (Fig. 3). The higher concentration of CNVs in telomeric regions aligns with previous studies in humans [49] and highlights how structural variations fuel further events by NAHR [48]. Finally, once arose in the bovine genome, CNVs can be inherited across generations [50], bringing out their potential application in breeding programs.

The distribution of detected CNV across chromosomes varies among dairy cattle populations, influenced by several factors, including breed, CNV calling method, genome assembly, SNP and CNV quality control, SNP array density, probe density in segmental duplication regions, and probe design [18, 21, 34, 51]. Hence, comparing CNVs between studies with diverse populations and methodologies is challenging, even within the same breed. This scenario underscores the need for integrating complementary methods and highlights the value of exploring CNV distribution and functionality across multiple populations. Previous studies have consistently shown that deletions are more frequent and smaller than duplications [10, 14, 50, 52], a finding also reported here. The reasons for the prevalence of deletions in the bovine genome remain unclear. However, it is known that the discovery of copy number deletions from SNP arrays is more sensitive than copy number duplications, contributing to higher frequencies and more precise breakpoint definitions for deletions than duplications. Moreover, the sensitivity of detecting CNV boundaries is directly related to the genome coverage, making the whole genome sequence (WGS) approaches preferable for in silico CNV identification. Currently, sequencing large animal numbers is limited due to the associated costs, generally restricting CNV analyses to a reduced number of animals, often to less than one hundred. Consequently, the entire population may be underrepresented, constraining CNVR identification and hampering its utilization in breeding programs.

The mapped CNVs were compiled into 1,184 CNVRs, covering 3.02% of the bovine autosome genome, coherent with other studies in Holsteins genotyped with high-density SNP arrays [10, 53]. Copy number variation regions summarize population CNV patterns and facilitate CNV functionality assessment. Notably, 82.34% of reported CNVRs overlapped by at least 50% in length with previously annotated structural variations in the bovine genome, confirming consistency with previous cattle CNV mapping studies and representativeness of the Holstein breed in our studied population. Additionally, we revealed 199 novel CNVRs overlapping functional genomic regions, including protein-coding genes and untranslated regions (UTRs). These new CNVRs overlapped genes like ALF Transcription Elongation Factor 1 (*AFF1*), DNA Meiotic Recombinase 1 (*DMC1*), Phospholipase D Family Member 4 (*PLD4*), Solute Carrier Family 39 Member 4 (*SLC39A4*), and TNF Receptor Superfamily Member 13B (*TNFRSF13B*), which play key roles in several biological processes. Interestingly, *AFF1* has been linked to heifer conception rate in U.S. Holsteins [54], whereas *DMC1* frameshift mutation causes nonobstructive azoospermia in humans [55]. Moreover, a nonsense mutation in *PLD4* and a splice variant in *SLC39A4* are responsible for bovine hereditary zinc deficiency in Fleckvieh and Holstein cattle, respectively [56, 57]. The *TNFRSF13B* gene regulates immune pathology resistance through innate B cell function in humans [58]. The high genomic coverage density and large population size enabled mapping new CNVs, encompassing genes underlying fertility and immunological mechanisms. Therefore, these findings provide novel insights into CNV influences on critical dairy cattle production traits.

Quantitative trait loci are genomic segments encompassing or linked to genes correlating with variation in polygenic traits. The CNVR-based QTL enrichment analysis reveals the strength of non-random overlap between QTLs and CNVRs. Thus, the smaller the *P*_FDR_, the stronger the evidence against randomness. This comprehensive CNVR-QTL screening identifies traits potentially affected by CNVs, providing evidence for future CNV-based genome-wide association studies. Notably, QTLs associated with milk, reproduction, health, production indices, exterior, carcass and meat traits overlapped with CNVRs beyond random chance in the studied population. Enriched QTLs were associated with 140 traits, including milk fat yield, milk yield, length of productive life, non-return rate, pregnancy rate, immunoglobulin G level, and net merit (Fig. 4). These traits directly impact milk production and quality, culling rate, reproductive performance, disease incidence, and overall productivity in dairy systems, highlighting the relevance of better understanding the CNV roles in biological processes. Indeed, previous studies have reported CNVs associated with breeding values for milk protein, milk fat, milk yield, somatic cell score, pregnancy rate, and net merit [8–10, 59]. Our findings align with these results, supporting the influence of CNVs on traits identified via QTL enrichment analysis. Although CNVs underline the immune response in dairy cattle [10, 17, 60], CNV-based GWAS on immune traits are lacking. Immunoglobulin G (IgG) concentration is an indicator of immune response, and CNVRs non-randomly overlapped QTLs associated with IgG level. Thus, plasma IgG concentration can be used efficiently to assess whether CNVs are linked to immune response in CNV-based GWAS. CNV-based GWAS captures genetic variation beyond traditional SNP-based GWAS by accounting for allele dosage. Studies have reported that some CNVs are in low linkage disequilibrium with SNPs [53], and roughly 25% of CNVs remain untagged by SNPs in Holsteins [61]. Furthermore, CNVs contribute to 18% of gene expression variation in humans [62]. Our QTL enrichment analysis suggests CNVs and QTLs segregate together and/or *de novo* CNV events are likely to occur in enriched QTLs associated with the aforementioned 140 complex traits.

Copy number variation regions overlapped with 2,788 genes, of which 87.34% are protein-coding genes. Genes are the most important DNA fragments responsible for encoding proteins essential for cellular activities. Markedly, 61.22% of overlapping genes were completely encompassed by CNVRs, potentially modifying their structure through changes in genic sequence and expression. In addition, CNVRs mapped onto stop-codon sequences can significantly alter the resulting protein by generating elongated transcripts. The remaining 38.78% of genes were partially overlapped with CNVRs, potentially affecting gene sequences through frameshift mutations and length changes, impacting mRNA sequences. Also, CNVRs overlapping non-coding intronic sequences of protein-coding genes can change gene length and expression levels, leading to under- or over-expression of affected or distant genes [64]. Consequently, copy number changes predominantly overlap genes, potentially modulating their expression at-locus or extra-locus, disrupting gene function, and contributing to phenotypic variation.

Copy number variation regions overlapped genes associated with cellular molecule transport mechanisms, health, growth, milk traits, and reproduction. Remarkably, CNVRs overlapped genes like ATP-binding cassette subfamily A member 9 (*ABCA9*), a member of the ABCA gene family responsible for regulating active transport in the placenta, which has been linked to abortion rate in Israeli dairy cattle [63]. Additionally, disintegrin and metalloproteinase with thrombospondin motifs gene family exhibits high expression in the embryonic subcutaneous fat and longissimus dorsi, particularly *ADAMTS13* has been associated with inflammatory response in bovine mammary epithelial cells [64]. A deletion CNVR encompassing 95 CNVs completely overlapped the Diacylglycerol O-Acyltransferase 1 (*DGAT1*) gene, known for its large effects on milk yield and composition [65], indicating that CNVs may underlie the genetic architecture of milk traits in Holstein cattle. The Guanylate Binding Protein 2 (*GBP2*) gene was tied to growth traits in Chinese cattle, potentially affecting skeletal muscle and fat development [66]. The homeobox gene family, including *HOXA5* and *HOXA9*, shows developmental stage-specific expression in bovine oocytes and early embryos [67], implying roles in regulating oocyte maturation and embryo development. CNVRs also overlapped with olfactory receptor family (e.g., *OR2A13* and *OR2L2*) known for the presence of copy number variants [68], and solute carrier gene family (e.g., *SLC3A2*) which is differentially expressed in the pregnant endometrium, facilitating maternal recognition of pregnancy that is critical for sustaining pregnancy in cattle [69].

The gene set analysis revealed overrepresented GO terms relevant to cattle health and reproductive traits. Notably, genes overlapping with CNVs and described in overrepresented GO terms include Defensin Beta 4 A (*DEFB4A*), GATA Binding Protein 3 (*GATA3*), Guanine Nucleotide-Binding Protein Subunit Beta-1 (*GNB1*), and Protein Phosphatase 1 Regulatory Inhibitor Subunit 1B (*PPP1R1B*). Remarkably, the “defense response” (GO:0006952) term highlights the *DEFB4A*, a β-Defensin gene known for antimicrobial activity against Gram-negative and Gram-positive bacteria and unicellular parasites [70]. β-Defensins are expressed in the mammary gland [71], potentially preventing early-stage intramammary infections. We identified high-confidence duplications (chr27:6684365–7186762) covering the entire *DEFB4A* gene (chr27:7138873–7140876) in 12 cows, implying increased *DEFB4A* copies potentially enhancing β-Defensins levels and immune response. The “signal transduction” term (GO:0007165) highlights *GATA3*, a key gene for maintaining the trophectoderm lineage in bovine embryos. Indeed, RNA-seq analysis shows that *GATA3* deletion disrupts the transcriptome in bovine blastocysts [72]. Importantly, we identified high-confidence deletions (chr13:15929813–15974589) overlapping 19.16% of the *GATA3* gene (chr13:15906719–15935286) in 9 cows, indicating possible implications of CNVs for pregnancy maintenance in Holsteins. In addition, the same “signal transduction” term encompasses the *GNB1* gene, essential for luteal sensitivity to PGF_2α_. In fact, *GNB1* expression increases over 10-fold in Prostaglandin F2 alpha (PGF_2α_)-treated D-4 corpus luteum compared to saline D-4 control in beef cattle [73], indicating the expression of *GNB1* was sensitive to exogenous PGF_2α_. We mapped high-confidence duplications (chr16:50871797–50917832) covering 59.70% of the *GNB1* gene (chr16:50856300–50933400) in 5 cows. Finally, the “signal transduction” term highlights the *PPP1R1B* gene, associated with subcutaneous fat deposition traits in Holstein cows and located near two QTLs linked with milk fatty acid content [74]. The *PPP1R1B* is involved in the cyclic adenosine monophosphate (cAMP) signaling pathway [75], known for regulating energetic metabolism with implications for development. Interestingly, we mapped high-confidence duplications (chr19: 39983228–40049807) covering 100% of the *PPP1R1B* gene (chr19:40006305–40015041) in 6 cows. Collectively, these findings emphasize the potential role of copy number variants in health and reproductive traits in dairy cattle.

In summary, we identified copy number variations in the bovine autosome genome using a large and representative high-density SNP-genotyped Holstein population and investigated their potential functional consequences by employing QTL and gene set enrichment analyses. This exploration revealed the nexuses between CNVs, quantitative traits, cellular and molecular functions, and biological processes. Notably, CNVs overlapped QTLs enriched for economically important traits in dairy cattle, including milk, reproduction, health, and production, emphasizing the importance and potential role of structural variations in breeding programs. The majority of mapped CNVRs overlapped genes, potentially influencing gene expression at-locus and/or distant genes by dosage changes or altered regulatory elements. The gene set analysis uncovered overrepresented GO terms related to metabolic processes, immune system, response to stimulus, and cellular binding activities. Therefore, our findings contribute to CNV annotation and characterization, suggesting that CNVs impact several economically relevant traits in dairy cattle.

## Conclusions

We mapped and characterized copy number variants in dairy cattle, revealing non-randomly overlaps with QTLs associated with milk, reproduction, and health traits. This suggests CNVs and QTLs may segregate together, impacting biological pathways underlying quantitative traits. Additionally, most of the CNVRs, including 199 newly reported ones, overlapped with genes, potentially modulating gene expression. Our findings indicate CNVs reside in functional genomic regions, impacting biological processes, molecular functions, and additive genetic variability. Therefore, this study provides a robust CNV map and functional insights, uncovering new copy number variants that warrant further exploration to better elucidate CNV roles in complex traits.

## Electronic supplementary material

Below is the link to the electronic supplementary material.

Supplementary Material 1

Supplementary Material 2

## Data Availability

All data analyzed during this study are public and/or included in this published article. See the supplementary materials. Mapped QTLs were reported by QTL ID numbers in release 50, available at the Cattle QTLdb (https://www.animalgenome.org/cgi-bin/QTLdb/BT/index). All previously mapped genes (https://ftp.ensembl.org/pub/release-109/gtf/bos_taurus/) and structural variations (https://ftp.ensembl.org/pub/release-109/variation/gvf/bos_taurus/) were retrieved from the ARS-USCD1.2 (release 109) available in the Ensembl database. The Variant Effect Prediction (VEP) analysis was performed using the Ensembl VEP (release 109) tool (https://useast.ensembl.org/info/docs/tools/vep/index.html). The gene set analysis was implemented using the overrepresentation test of the PANTHER software v19.0 (https://www.pantherdb.org/). The accession code for the genotypes of this Holstein population was made available in Seabury et al. (2023) (https://datadryad.org/dataset/doi:10.5061/dryad.0gb5mkm04).

## References

[1] USDA, Milk. Mar: Production per cow by year 2014–2023, US. https://www.nass.usda.gov/Charts_and_Maps/Milk_Production_and_Milk_Cows/cowrates.php. Accessed 11 2024.

[2] USDA. Milk cows: Inventory by year 2014–2023, US. https://www.nass.usda.gov/Charts_and_Maps/Milk_Production_and_Milk_Cows/milkcows.php. Accessed 11 Mar 2024.

[3] CDCB. Genetic Trend. https://webconnect.uscdcb.com/#/summary-stats/genetic-trend. Accessed 31 Oct 2024.

[4] Guinan FL, Wiggans GR, Norman HD, Dürr JW, Cole JB, Van Tassell CP, et al. Changes in genetic trends in US dairy cattle since the implementation of genomic selection. J Dairy Sci. 2023;106:1110–29. 36494224 10.3168/jds.2022-22205

[5] Feuk L, Carson AR, Scherer SW. Structural variation in the human genome. Nat Rev Genet. 2006;7:85–97. 16418744 10.1038/nrg1767

[6] Henrichsen CN, Vinckenbosch N, Zöllner S, Chaignat E, Pradervand S, Schütz F, et al. Segmental copy number variation shapes tissue transcriptomes. Nat Genet. 2009;41:424–9. 19270705 10.1038/ng.345

[7] Zhang F, Gu W, Hurles ME, Lupski JR. Copy number variation in human health, disease, and evolution. Annu Rev Genomics Hum Genet. 2009;10:451–81. 19715442 10.1146/annurev.genom.9.081307.164217 PMC4472309

[8] Zhou Y, Connor EE, Wiggans GR, Lu Y, Tempelman RJ, Schroeder SG, et al. Genome-wide copy number variant analysis reveals variants associated with 10 diverse production traits in Holstein cattle. BMC Genomics. 2018;19:314. 29716533 10.1186/s12864-018-4699-5 PMC5930521

[9] Gao Y, Jiang J, Yang S, Hou Y, Liu GE, Zhang S, et al. CNV discovery for milk composition traits in dairy cattle using whole genome resequencing. BMC Genomics. 2017;18:1–12. 28356085 10.1186/s12864-017-3636-3 PMC5371188

[10] Aguilar MD, Ponce SIR, López FJR, Padilla EG, Peláez CGV, Bagnato A, et al. Genome-wide association study for milk somatic cell score in Holstein cattle using copy number variation as markers. J Anim Breed Genet. 2017;134:49–59. 27578198 10.1111/jbg.12238

[11] Lee YL, Takeda H, Moreira GCM, Karim L, Mullaart E, Coppieters W, et al. A 12 kb multi-allelic copy number variation encompassing a GC gene enhancer is associated with mastitis resistance in dairy cattle. PLoS Genet. 2021;17:7. 10.1371/journal.pgen.1009331 PMC8328317 34288907

[12] Sassi NB, González-Recio Ó, Río RPD, Rodríguez-Ramilo ST, Fernández AI. Associated effects of copy number variants on economically important traits in Spanish Holstein dairy cattle. J Dairy Sci. 2016;99:6371–80. 27209136 10.3168/jds.2015-10487

[13] Ma L, Chung WK. Quantitative analysis of copy number variants based on real-time lightcycler PCR. Curr Protoc Hum Genet. 2014;80:7. 10.1002/0471142905.hg0721s80 PMC3949243 24510682

[14] Mielczarek M, Frąszczak M, Giannico R, Minozzi G, Williams JL, Wojdak-Maksymiec K, et al. Analysis of copy number variations in Holstein-Friesian cow genomes based on whole-genome sequence data. J Dairy Sci. 2017;100:5515–25. 28501396 10.3168/jds.2016-11987

[15] Keel BN, Lindholm-Perry AK, Snelling WM. Evolutionary and functional features of copy number variation in the cattle genome. Front Genet. 2016;7:207. 27920798 10.3389/fgene.2016.00207 PMC5118444

[16] Choi J-W, Chung W-H, Lim K-S, Lim W-J, Choi B-H, Lee S-H, et al. Copy number variations in Hanwoo and Yanbian cattle genomes using the massively parallel sequencing data. Gene. 2016;589:36–42. 27188257 10.1016/j.gene.2016.05.017

[17] Braga LG, Chud TCS, Watanabe RN, Savegnago RP, Sena TM, Carmo AS, et al. Identification of copy number variations in the genome of dairy Gir cattle. PLoS ONE. 2023;18:4. 10.1371/journal.pone.0284085 PMC10085049 37036840

[18] Winchester L, Yau C, Ragoussis J. Comparing CNV detection methods for SNP arrays. Brief Funct Genomic Proteomic. 2009;8:353–66. 19737800 10.1093/bfgp/elp017

[19] Duan J, Zhang J-G, Deng H-W, Wang Y-P. Comparative studies of copy number variation detection methods for Next-Generation sequencing technologies. PLoS ONE. 2013;8:3. 10.1371/journal.pone.0059128 PMC3604020 23527109

[20] Zhang X, Du R, Li S, Zhang F, Jin L, Wang H. Evaluation of copy number variation detection for a SNP array platform. BMC Bioinformatics. 2014;15:50. 24555668 10.1186/1471-2105-15-50 PMC4015297

[21] Xu L, Hou Y, Bickhart D, Song J, Liu G. Comparative analysis of CNV calling algorithms: literature survey and a case study using bovine High-Density SNP data. Microarrays. 2013;2:171–85. 27605188 10.3390/microarrays2030171 PMC5003459

[22] Zhao M, Wang Q, Wang Q, Jia P, Zhao Z. Computational tools for copy number variation (CNV) detection using next-generation sequencing data: features and perspectives. BMC Bioinformatics. 2013;14:1–16. 24564169 10.1186/1471-2105-14-S11-S1 PMC3846878

[23] Salomón-Torres R, González-Vizcarra VM, Medina-Basulto GE, Montaño-Gómez MF, Mahadevan P, Yaurima-Basaldúa VH, et al. Genome-wide identification of copy number variations in Holstein cattle from Baja California, Mexico, using high-density SNP genotyping arrays. Genet Mol Res. 2015;14:11848–59. 26436509 10.4238/2015.October.2.18

[24] Upadhyay M, da Silva VH, Megens H-J, Visker MHPW, Ajmone-Marsan P, Bâlteanu VA, et al. Distribution and functionality of copy number variation across European cattle populations. Front Genet. 2017;8:23. 28878807 10.3389/fgene.2017.00108 PMC5572341

[25] Goyache F, Pérez-Pardal L, Fernández I, Traoré A, Menéndez-Arias NA, Arias KD, et al. Identification and characterization of copy number variations regions in West African taurine cattle. Animals. 2022;12:2130. 36009719 10.3390/ani12162130 PMC9405125

[26] Lemos MVA, Berton MP, Camargo GMF, Peripolli E, Silva RMO, Olivieri BF, et al. Copy number variation regions in Nellore cattle: evidences of environment adaptation. Livest Sci. 2018;207:51–8.

[27] Butty AM, Chud TCS, Miglior F, Schenkel FS, Kommadath A, Krivushin K, et al. High confidence copy number variants identified in Holstein dairy cattle from whole genome sequence and genotype array data. Sci Rep. 2020;10:8044. 32415111 10.1038/s41598-020-64680-3 PMC7229195

[28] Pinedo P, Santos JEP, Chebel RC, Galvão KN, Schuenemann GM, Bicalho RC, et al. Associations of reproductive indices with fertility outcomes, milk yield, and survival in Holstein cows. J Dairy Sci. 2020;103:6647–60. 32359989 10.3168/jds.2019-17867

[29] Lopes F, Rosa G, Pinedo P, Santos JEP, Chebel RC, Galvao KN, et al. Genome-enable prediction for health traits using high-density SNP panel in US Holstein cattle. Anim Genet. 2020;51:192–9. 31909828 10.1111/age.12892 PMC7065151

[30] Seabury CM, Smith JL, Wilson ML, Bhattarai E, Santos JEP, Chebel RC, et al. Genome-wide association and genomic prediction for a reproductive index summarizing fertility outcomes in U.S. Holsteins. G3. 2023;13:9. 10.1093/g3journal/jkad043 PMC10468724 36848195

[31] Zimin AV, Delcher AL, Florea L, Kelley DR, Schatz MC, Puiu D, et al. A whole-genome assembly of the domestic cow, Bos Taurus. Genome Biol. 2009;10:1–10. 10.1186/gb-2009-10-4-r42 PMC2688933 19393038

[32] Rosen BD, Bickhart DM, Schnabel RD, Koren S, Elsik CG, Tseng E, et al. De Novo assembly of the cattle reference genome with single-molecule sequencing. Giga Sci. 2020;9:1–9. 10.1093/gigascience/giaa021 PMC7081964 32191811

[33] Masuda Y. User’s Manual for QCF90. 2020. Available at http://nce.ads.uga.edu/wiki/lib/exe/fetch.php?media=pdf:manual_qc.pdf. Accessed 20 Oct 2023.

[34] Wang K, Li M, Hadley D, Liu R, Glessner J, Grant SFA, et al. PennCNV: an integrated hidden Markov model designed for high-resolution copy number variation detection in whole-genome SNP genotyping data. Genome Res. 2007;17:1665–74. 17921354 10.1101/gr.6861907 PMC2045149

[35] Silva V, Ramos M, Groenen M, Crooijmans R, Johansson A, Regitano L, et al. CNVRanger: association analysis of CNVs with gene expression and quantitative phenotypes. Bioinformatics. 2020;36:972–3. 31392308 10.1093/bioinformatics/btz632 PMC9887538

[36] Wickham H. ggplot2: Elegant Graphics for Data Analysis. 2016. https://ggplot2-book.org/. Accessed 6 Dec 2023.

[37] Harrison PW, Amode MR, Austine-Orimoloye O, Azov AG, Barba M, Barnes I, et al. Ensembl 2024. Nucleic Acids Res. 2024;52:891–9. 10.1093/nar/gkad1049 PMC10767893 37953337

[38] Hu Z-L, Park CA, Reecy JM. Bringing the animal QTLdb and CorrDB into the future: meeting new challenges and providing updated services. Nucleic Acids Res. 2022;50:956–61. 10.1093/nar/gkab1116 PMC8728226 34850103

[39] Fisher RA. On the interpretation of Χ 2 from contingency tables, and the calculation of P. J R Statist Soc. 1922;85:87.

[40] Tavazoie S, Hughes JD, Campbell MJ, Cho RJ, Church GM. Systematic determination of genetic network architecture. Nat Genet. 1999;22:281–5. 10391217 10.1038/10343

[41] Benjamini Y, Hochberg Y. Controlling the false discovery rate: A practical and powerful approach to multiple testing. J R Statist Soc. 1995;57:289–300.

[42] McLaren W, Gil L, Hunt SE, Riat HS, Ritchie GRS, Thormann A, et al. The ensembl variant effect predictor. Genome Biol. 2016;17:1–14. 27268795 10.1186/s13059-016-0974-4 PMC4893825

[43] Thomas PD, Ebert D, Muruganujan A, Mushayahama T, Albou LP, Mi H. PANTHER: making genome-scale phylogenetics accessible to all. Prot Sci. 2022;31:8–22. 10.1002/pro.4218 PMC8740835 34717010

[44] Mi H, Muruganujan A, Huang X, Ebert D, Mills C, Guo X, et al. Protocol update for large-scale genome and gene function analysis with the PANTHER classification system. Nat Protoc. 2019;14:3:703–21. 30804569 10.1038/s41596-019-0128-8 PMC6519457

[45] Stankiewicz P, Lupski JR. Structural variation in the human genome and its role in disease. Annu Rev Med. 2010;61:437–55. 20059347 10.1146/annurev-med-100708-204735

[46] Gu W, Zhang F, Lupski JR. Mechanisms for human genomic rearrangements. Pathogenetics. 2008;1:4. 19014668 10.1186/1755-8417-1-4 PMC2583991

[47] Hastings PJ, Lupski JR, Rosenberg SM, Ira G. Mechanisms of change in gene copy number. Nat Rev Genet. 2009;10:551–64. 19597530 10.1038/nrg2593 PMC2864001

[48] Lupski JR. Genomic disorders: structural features of the genome can lead to DNA rearrangements and human disease traits. Trends Genet. 1998;14:417–22. 9820031 10.1016/s0168-9525(98)01555-8

[49] Nguyen D-Q, Webber C, Ponting CP. Bias of selection on human Copy-Number variants. PLoS Genet. 2006;2:2. 10.1371/journal.pgen.0020020 PMC1366494 16482228

[50] Hou Y, Liu GE, Bickhart DM, Cardone MF, Wang K, Kim E, et al. Genomic characteristics of cattle copy number variations. BMC Genomics. 2011;12:1–11. 10.1186/1471-2164-12-127 PMC3053260 21345189

[51] Redon R, Ishikawa S, Fitch KR, Feuk L, Perry GH, Andrews TD, et al. Global variation in copy number in the human genome. Nature. 2006;444:444–54. 17122850 10.1038/nature05329 PMC2669898

[52] Butty AM, Chud TCS, Cardoso DF, Lopes LSF, Miglior F, Schenkel FS, et al. Genome-wide association study between copy number variants and hoof health traits in Holstein dairy cattle. J Dairy Sci. 2021;104:8050–61. 33896633 10.3168/jds.2020-19879

[53] Lee YL, Bosse M, Mullaart E, Groenen MAM, Veerkamp RF, Bouwman AC. Functional and population genetic features of copy number variations in two dairy cattle populations. BMC Genomics. 2020;21:89. 31992181 10.1186/s12864-020-6496-1 PMC6988284

[54] Jiang J, Ma L, Prakapenka D, VanRaden PM, Cole JB, Da Y. A Large-Scale Genome-Wide association study in U.S. Holstein cattle. Front Genet. 2019;10:412. 31139206 10.3389/fgene.2019.00412 PMC6527781

[55] Cao D, Shi F, Guo C, Liu Y, Lin Z, Zhang J, et al. A pathogenic DMC1 frameshift mutation causes nonobstructive azoospermia but not primary ovarian insufficiency in humans. Mol Hum Reprod. 2021;27:9. 10.1093/molehr/gaab058 34515795

[56] Jung S, Pausch H, Langenmayer MC, Schwarzenbacher H, Majzoub-Altweck M, Gollnick NS, et al. A nonsense mutation in PLD4 is associated with a zinc deficiency-like syndrome in Fleckvieh cattle. BMC Genomics. 2014;15:623. 25052073 10.1186/1471-2164-15-623 PMC4117962

[57] Yuzbasiyan-Gurkan V, Bartlett E. Identification of a unique splice site variant in SLC39A4 in bovine hereditary zinc deficiency, lethal trait A46: an animal model of acrodermatitis enteropathica. Genomics. 2006;88:521–6. 16714095 10.1016/j.ygeno.2006.03.018

[58] Barbosa MGM, Lefferts AR, Huynh D, Liu H, Zhang Y, Fu B, et al. TNFRSF13B genotypes control immune-mediated pathology by regulating the functions of innate B cells. JCI Insight. 2021;6:17. 10.1172/jci.insight.150483 PMC8492324 34283811

[59] Liu M, Fang L, Liu S, Pan MG, Seroussi E, Cole JB, et al. Array CGH-based detection of CNV regions and their potential association with reproduction and other economic traits in Holsteins. BMC Genomics. 2019;20:181. 30845913 10.1186/s12864-019-5552-1 PMC6407259

[60] Hou Y, Bickhart DM, Chung H, Hutchison JL, Norman HD, Connor EE, et al. Analysis of copy number variations in Holstein cows identify potential mechanisms contributing to differences in residual feed intake. Funct Integr Genomics. 2012;12:717–23. 22991089 10.1007/s10142-012-0295-y

[61] Xu L, Cole JB, Bickhart DM, Hou Y, Song J, VanRaden PM, et al. Genome wide CNV analysis reveals additional variants associated with milk production traits in Holsteins. BMC Genomics. 2014;15:1–10. 25128478 10.1186/1471-2164-15-683 PMC4152564

[62] Stranger BE, Forrest MS, Dunning M, Ingle CE, Beazlsy C, Thorne N, Relative impact of nucleotide and copy number variation on gene phenotypes. Science., Juan D, Valencia A, Rico D et al. Intronic CNVs and gene expression variation in human populations. PLoS Genet. 2019;15:e1007902. 10.1371/journal.pgen.1007902 PMC6345438 30677042

[63] Gershoni M, Ezra E, Weller JI. Genetic and genomic analysis of long insemination interval in Israeli dairy cattle as an indicator of early abortions. J Dairy Sci. 2020;103:4495–509. 32113774 10.3168/jds.2019-17482

[64] Sheng H, Zhang J, Pan C, Wang S, Gu S, Li F, et al. Genome-wide identification of bovine ADAMTS gene family and analysis of its expression profile in the inflammatory process of mammary epithelial cells. Int J Biol Macromol. 2023;244:125304. 37315674 10.1016/j.ijbiomac.2023.125304

[65] Grisart B, Coppieters W, Farnir F, Karim L, Ford C, Berzi P, et al. Positional candidate cloning of a QTL in dairy cattle: identification of a missense mutation in the bovine DGAT1 gene with major effect on milk yield and composition. Genome Res. 2001;12:222–31. 10.1101/gr.224202 11827942

[66] Zhang GM, Zheng L, He H, Song CC, Zhang ZJ, Cao XK, et al. Associations of GBP2 gene copy number variations with growth traits and transcriptional expression in Chinese cattle. Gene. 2018;647:101–6. 29325733 10.1016/j.gene.2018.01.004

[67] Paul D, Bridoux L, Rezsöhazy R, Donnay I. HOX genes are expressed in bovine and mouse oocytes and early embryos. Mol Reprod Dev. 2011;78:436–49. 21567651 10.1002/mrd.21321

[68] Lee K, Nguyen DT, Choi M, Cha SY, Kim JH, Dadi H, et al. Analysis of cattle olfactory subgenome: the first detail study on the characteristics of the complete olfactory receptor repertoire of a ruminant. BMC Genomics. 2013;14:596. 24004971 10.1186/1471-2164-14-596 PMC3766653

[69] Adhikari B, Lee CN, Khadka VS, Deng Y, Fukumoto G, Thorne M, et al. RNA-Sequencing based analysis of bovine endometrium during the maternal recognition of pregnancy. BMC Genomics. 2022;23:1–15. 35799127 10.1186/s12864-022-08720-4 PMC9264496

[70] Brogden KA. Antimicrobial peptides: pore formers or metabolic inhibitors in bacteria? Nat Rev Microbiol. 2005;3:238–50. 15703760 10.1038/nrmicro1098

[71] Tetens J, Friedrich JJ, Hartmann A, Schwerin M, Kalm E, Thaller G. The Spatial expression pattern of antimicrobial peptides across the healthy bovine udder. J Dairy Sci. 2010;93:775–83. 20105549 10.3168/jds.2009-2729

[72] Shi Y, Hu B, Wang Z, Wu X, Luo L, Li S, et al. Functional role of GATA3 and CDX2 in lineage specification during bovine early embryonic development. Reproduction. 2023;165:325–33. 36630554 10.1530/REP-22-0269 PMC9986393

[73] Goravanahally MP, Salem M, Yao J, Inskeep EK, Flores JA. Differential gene expression in the bovine corpus luteum during transition from early phase to midphase and its potential role in acquisition of luteolytic sensitivity to prostaglandin F2 Alpha1. Biol Reprod. 2009;80:980–8. 19164179 10.1095/biolreprod.108.069518

[74] Zhang H, Mi S, Brito LF, Hu L, Wang L, Ma L, et al. Genomic and transcriptomic analyses enable the identification of important genes associated with subcutaneous fat deposition in Holstein cows. J Genet Genomics. 2023;50:385–97. 36738887 10.1016/j.jgg.2023.01.011

[75] Kotecha S, Lebot MN, Sukkarn B, Ball G, Moseley PM, Chan SY, et al. Dopamine and cAMP-regulated phosphoprotein 32 kda (DARPP-32) and survival in breast cancer: a retrospective analysis of protein and mRNA expression. Sci Rep. 2019;9:16987. 31740718 10.1038/s41598-019-53529-z PMC6861271

