# Supplementary material for: Detecting and characterizing copy number variation in a large commercial U.S. Holstein cattle population
Source: BMC Genomics. 2025 Apr 16;26:381. doi: 10.1186/s12864-025-11536-7 (PMC12004798; doi:10.1186/s12864-025-11536-7)
Supplement: Supplementary file 1 — Supplementary Material 1 [file 12864_2025_11536_MOESM1_ESM.docx]

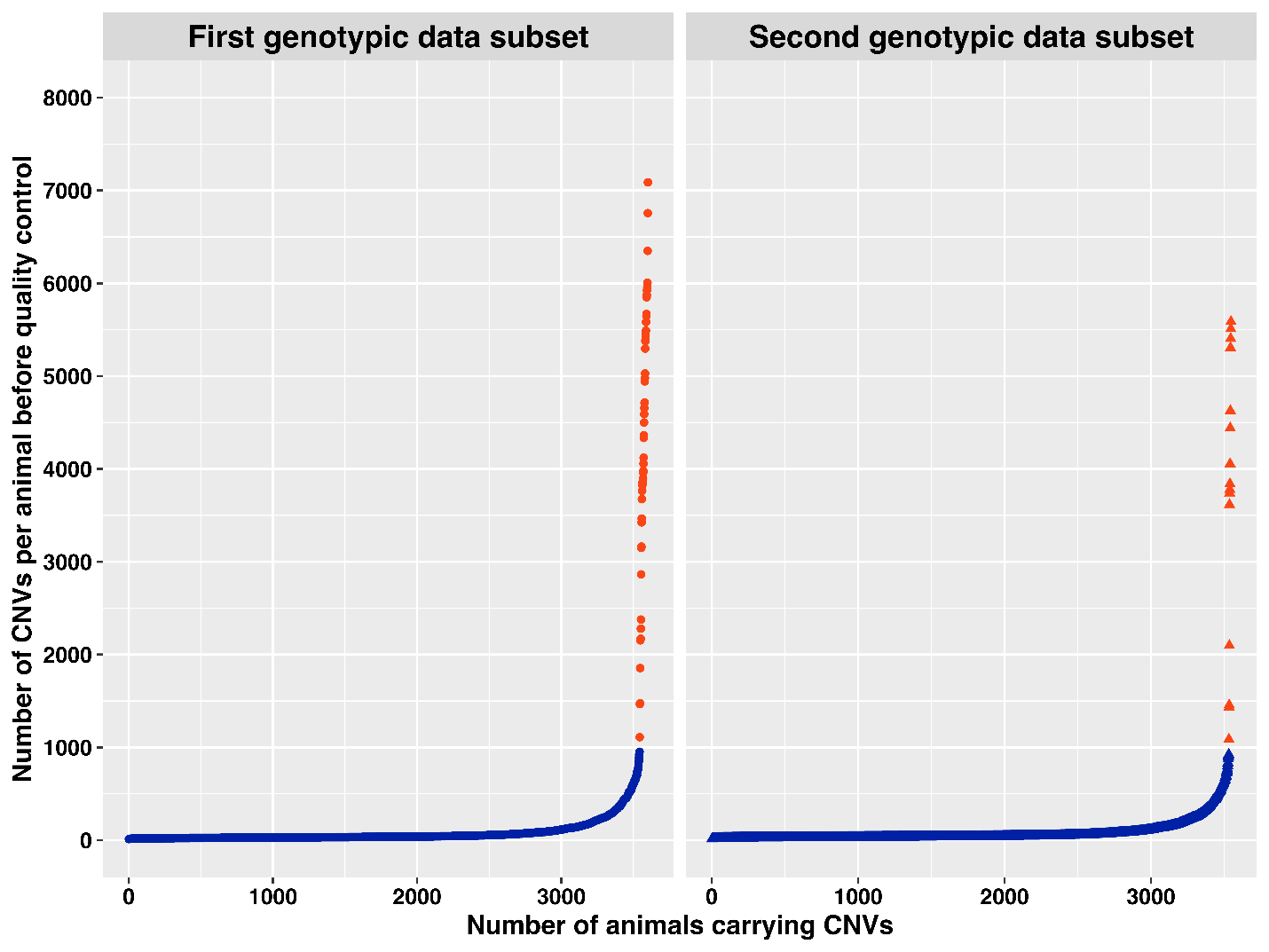


**Figure S1. Copy number variation counts per animal from two genotypic data subsets prior to the three-step quality control.** The first subset (dots; 3,601 animals) lacked animal and SNP quality control prior to CNV calling, while the second (triangles; 3,546 animals) included solely samples and SNPs with ≥90% call rates. Given the CNV count in the population under study, outliers (orange; 58 and 15 animals) were defined as animals with more than 1,000 CNVs, indicating possible low sample quality, in contrast to animals with ≤1,000 CNVs (blue; 3,543 and 3,531 animals, respectively).


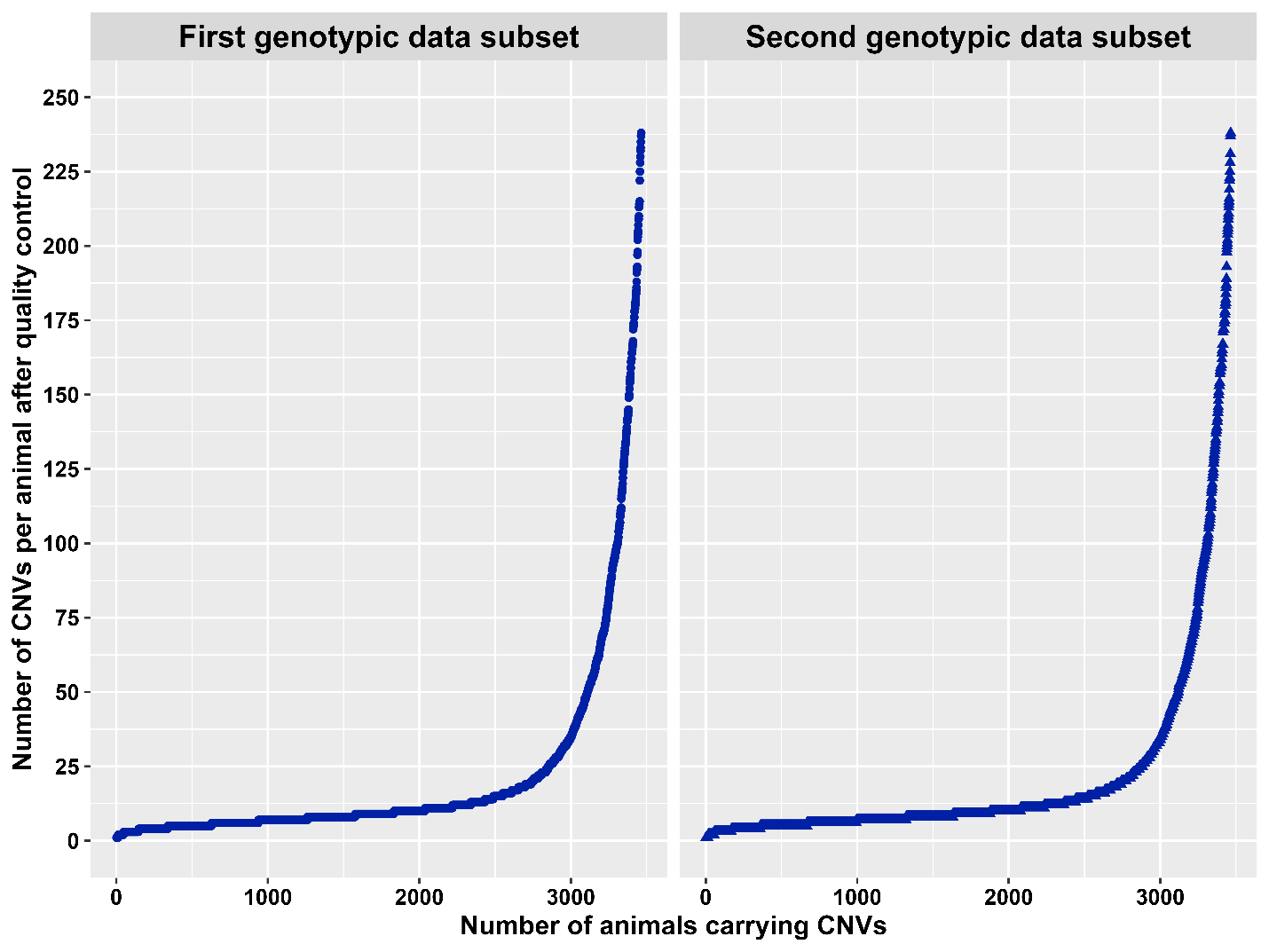
**Figure S2.** **Copy number variation counts per animal from two genotypic data subsets after the three-step quality control.** Criteria for keeping animals included LRR SD ≤ 0.30, BAF drift ≤ 0.01, waviness factor ≤ 0.05, and CNV count ≤ 1,000. Structural variation filtering required at least 10 SNPs and 1 kb length, and presence in a minimum of 5 animals, retaining 3,465 animals with a maximum of 238 CNVs per animal in both subsets.

**
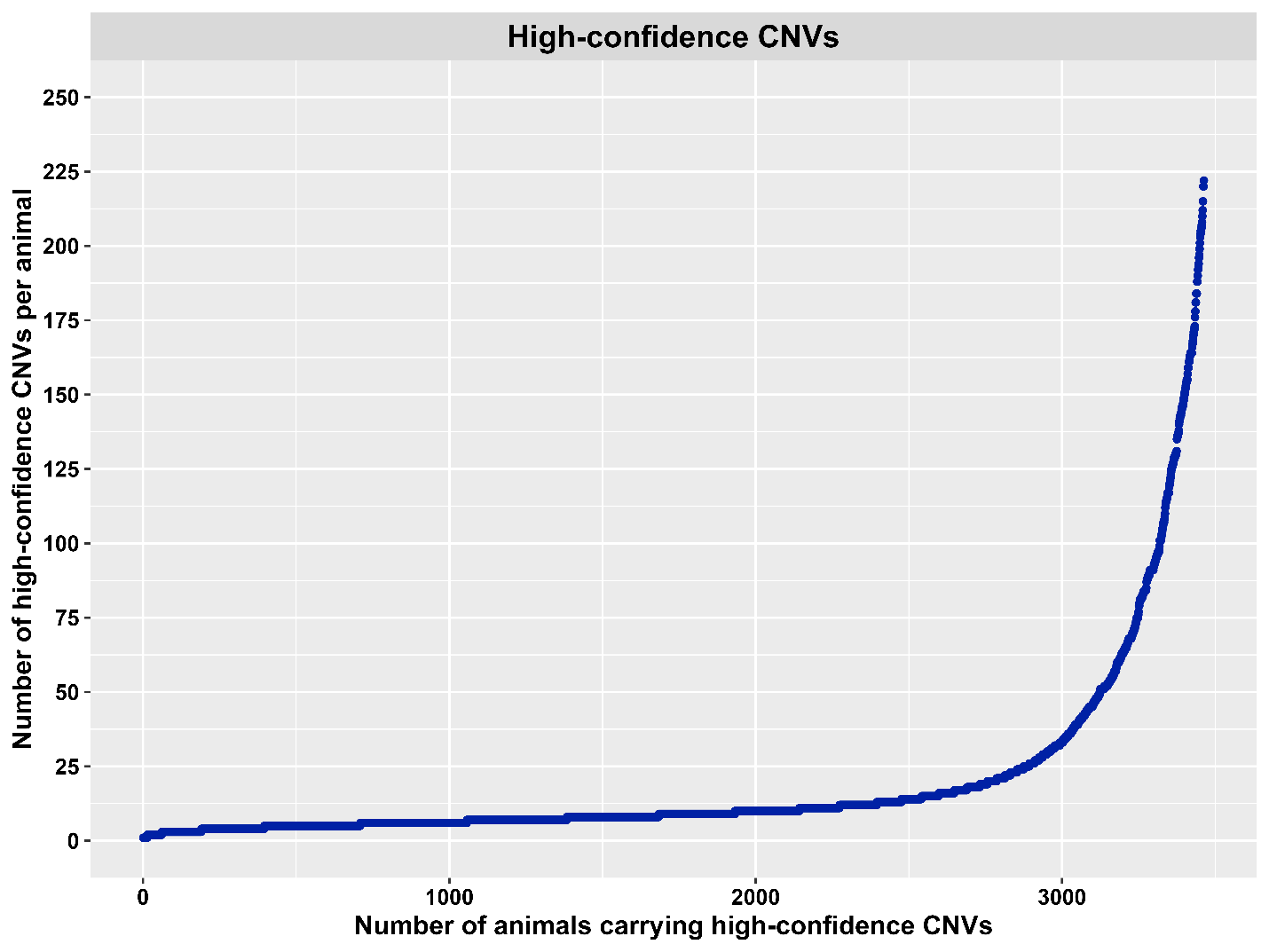
Figure S3. High-confidence CNV counts per animal.** A total of 3,463 animals with a maximum of 222 high-confidence CNV counts per animal (68,982 in total). High-confidence CNVs were considered for further analysis. Interestingly, two animals from each genotypic subset had CNVs absent in the other subset. Only CNVs identified in both subsets after the three-step quality control were classified as high-confidence, which reduced CNV counts compared to the first and second genotypic subsets.

**
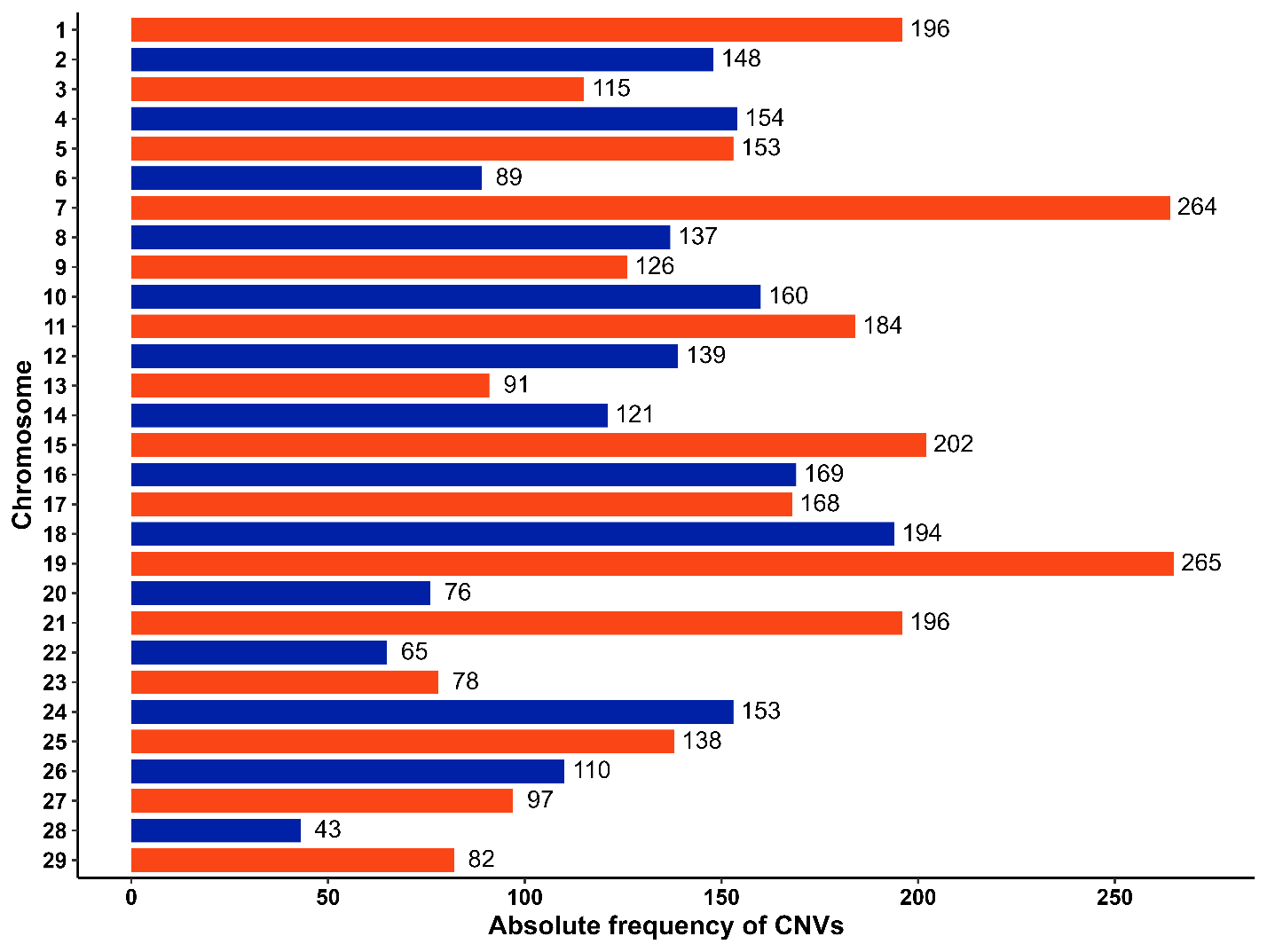
**

**Figure S4. Absolute frequency of non-redundant high-confidence CNVs per chromosome.** On average 141.82 non-redundant CNVs were mapped per chromosome, ranging from 43 CNVs on BTA 28 to 265 CNVs on BTA 19.
